# Geochemistry of soil gas in the seismic fault zone produced by the Wenchuan *M*s 8.0 earthquake, southwestern China

**DOI:** 10.1186/1467-4866-11-5

**Published:** 2010-12-06

**Authors:** Xiaocheng Zhou, Jianguo Du, Zhi Chen, Jianwu Cheng, Yi Tang, Liming Yang, Chao Xie, Yueju Cui, Lei Liu, Li Yi, Panxin Yang, Ying Li

**Affiliations:** 1 School of Earth and Space Sciences, University of Science and Technology of China, Hefei 230026, China; 2 Institute of Earthquake Science, China Earthquake Administration, Beijing 100036, China; 3 Earthquake Administration of Gansu Province, Lanzhou 730000, China

## Abstract

The spatio-temporal variations of soil gas in the seismic fault zone produced by the 12 May 2008 Wenchuan *M*s 8.0 earthquake were investigated based on the field measurements of soil gas concentrations after the main shock. Concentrations of He, H_2_, CO_2_, CH_4_, O_2_, N_2_, Rn, and Hg in soil gas were measured in the field at eight short profiles across the seismic rupture zone in June and December 2008 and July 2009. Soil-gas concentrations of more than 800 sampling sites were obtained. The data showed that the magnitudes of the He and H_2 _anomalies of three surveys declined significantly with decreasing strength of the aftershocks with time. The maximum concentrations of He and H_2 _(40 and 279.4 ppm, respectively) were found in three replicates at the south part of the rupture zone close to the epicenter. The spatio-temporal variations of CO_2_, Rn, and Hg concentrations differed obviously between the north and south parts of the fault zone. The maximum He and H_2 _concentrations in Jun 2008 occurred near the parts of the rupture zone where vertical displacements were larger. The anomalies of He, H_2_, CO_2_, Rn, and Hg concentrations could be related to the variation in the regional stress field and the aftershock activity.

## 1. Introduction

The *M*_S _8.0 Wenchuan earthquake of 12 May 2008 produced a 285-km long surface rupture zone along the pre-existing Yingxiu-Beichuan, Guanxian-Anxian, and Qingchuan faults, which indicated the faults were reactivated. The maximum thrust slip distance was estimated to be about 10 m, accompanied by 9 m of shortening across the rupture zone [1]. Most of the large aftershocks were distributed in the north and south parts of the Longmenshan fault zone. The dominant focal depths of the aftershocks were between 5 and 20 km, and the depth of the main shock was 16.0 km. The focal depth distribution in some areas was characterized by high-angle westward dipping. The rupture mode of the main shock was characterized by reverse faulting in the south and with a large strike-slip component in the north [2].

The spatial and temporal patterns of deep-source gas leakage can be investigated by measuring soil gas in the faulted zones. The variations of soil-gas concentrations can serve as useful tools for monitoring earthquakes. The short-term decrease of helium concentrations in soil gas along the San Andreas Fault in central California was well-correlated with aftershocks [3]. The sudden increase in CO_2 _and Rn concentrations at the Mariánské Láznĕ fault in the NW Bohemian swarm quake region might indicate an increase of fault permeability caused by stress redistribution, resulting in the opening of migration pathways [4]. Across two fault segments that ruptured during the magnitude 7.5 Landers earthquake in 1992, anomalously high radon concentrations were found in the fractures three weeks after the earthquake [5]. In parts of southeastern Ghana, In the highly faulted area, radon activity up to 115.00 k Bq/m^3 ^was measured, on the contrary in non-faulted areas radon activity was less than 20.00 k Bq m^3 ^[6]. The concentrations of He, Rn, CO_2_, and N_2 _in soil gas clearly showed anomalous values along the fault of Hsincheng in the Hsinchu area of Taiwan [7]. High H_2 _concentrations of up to 3% were observed in soil gas along a strike of the Yamasaki fault [8].

The compositions and distributions of soil gases in a fault zone are affected by many factors, such as agrologic, biogenic, and meteorological factors, especially gas leakage from deep faults [9]. Therefore, the anomaly variation of soil gas concentrations induced by the earthquake is difficult to distinguish. The goals of this paper are to correlate the spatio-temporal variations of soil gas along the seismic rupture zone of the Wenchuan *M*s 8.0 earthquake with the aftershocks and to try to couple the geochemical field with the crustal stress field.

## 2. Geological setting

The Longmenshan fault zone (LFZ) consists of the Maoxian-Wenchuan fault, the Qingchuan fault, the Beichuan-Yingxiu fault, and the Penxian-Guanxian fault from west to east, and is located at the east margin of the Qinghai-Tibetan Plateau. These faults with a northeast-southwest strike began to develop in the Triassic. Due to episodic movements during the Meso-Cenozoic, especially since the Late Cenozoic, accompanied by eastward extrusion of the Qinghai-Tibet Plateau, the middle and southern segments of the LFZ underwent strong compression and the basement was detached, which formed impressive thrust nappe structures and klippen swarms on the eastern margin of the Qinghai-Tibet Plateau [10]. The LFZ penetrates the Moho. Strata in the fault zone are predominantly Palaeozoic and Lower Mesozoic flysch and carbonate, with some intermediate layers of volcanic rocks. Many great earthquakes have been recorded along the Longmenshan fault zone. For instance, two *M*s 7.2 earthquakes occurred between Songpan and Pingwu, north of Sichuan Province on August 16 and 23, 1976. An *Ms *7.5 earthquake occurred at Diexi town, Maoxian county, Sichuan Province on August 25, 1933 [11].

## 3. Methods

**Figure 1:**
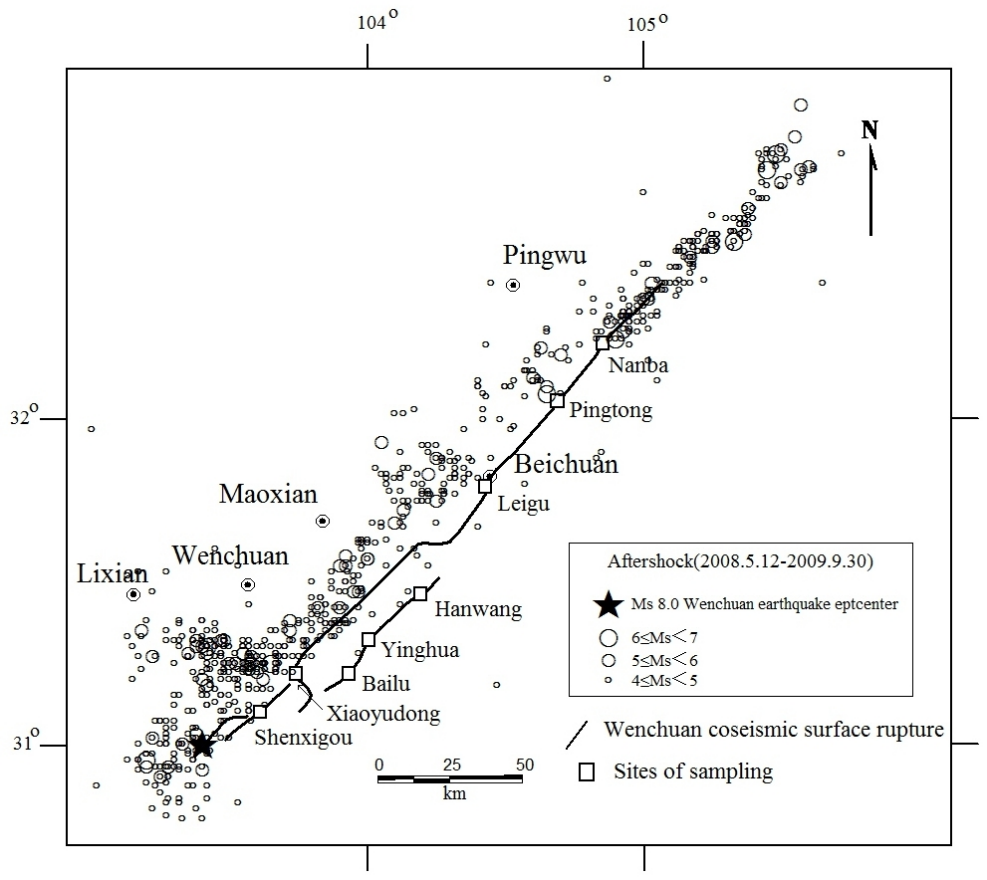
**Location of sampling sites in the seismic fault zone produced by Wenchuan *M*s 8.0 earthquake**.

Soil-gas concentrations were repeatedly measured with a time interval of a half year in the field along the profiles approximately perpendicular to the fault scarps produced by the *Ms *8.0 Wenchuan earthquake. The locations of sampling sites are followed: 1. Shenxigou Village of Hongkou town (SXG, N31°05.363', E103°36.895'), 2.Luoyuan Village of Xiaoyudong Town(XYD, N31°11.699', E103°45.228'), 3. The centre school of Bailu Town (BL, N31°12.667', E103°54.735'), 4.Yinghua Town (YH, N31°19.168', E104°00.085'), 5.Quanxin Village of Hanwang Town (HW, N31°28.694', E104°12.215'), 6.Shiyan Village of Leigu Town (LG, N31°46.808', E104°25.220'), 7.Pingtong Town (PT, N32°03.902', E104°41.448'), 8.Miaoziwan of Nanba Town (NB, N32°12.416', E104°50.659')(Figure 1).

The first soil-gas survey was performed from 20 Jun to 3 Jul in 2008; 196 samples were measured at five sites (SXG, BL, YH, LG and PT, Figure 1). The second and third surveys were carried out at eight sites (SXG, XYD, BL, YH, HW, LG and PT, NB, Figure 1) in the same areas from 16 to 31 Dec in 2008 and from 16 Jul to 31 Aug in 2009, and 331 and 301 samples were measured, respectively. Three or four parallel profiles at each of eight sampling sites were analyzed. Soil gas was sampled along profiles at distances of about 5 to 20 m, and the distances were shortened to 2.5 m near the fault scarps. He, H_2_, N_2_, O_2_, CO_2_, CH_4_, Rn, and Hg concentrations of 828 soil-gas samples were analyzed.

The traditional method was employed to sample soil gases [12]. Three holes strike of 0.8 m deep and 2.3 cm in diameter at 0.5 m lateral intervals were drilled in the ground at each sampling site. Three Teflon tube probes strike of 80 cm long and 2.3 cm in diameter were inserted into the holes. The concentration of elemental mercury in the soil was analyzed in the field with a portable RA-915+ Zeeman Mercury Analyzer and the error of measurement was 2 ng/m^3^. The soil gas from two holes was continuously pumped through the analyzer cell at a rate of 2 L/min. Data were recorded on the portable computer with a response time of 1 sec. ^222^Rn concentration was measured in the field using a RAD7 Radon Detector. Soil gas was pumped at flow rate of 1 L/min from another probe into the internal accumulation chamber where electrostatic collection of alpha-emitters with spectral analysis took place. Alpha emissions with energy of 6.002 MeV were attributed to ^218^Po decay, allowing ^222^Rn activity to be produced [13]. Three concentrations of radon were performed each with 5 minute integration time. The results in units of Bq/m^3 ^were determined by the mean value of three measurements with an error of ± 5%. The concentrations of He, H_2_, N_2_, O_2_, CO_2 _and CH_4 _in soil gas were measured in the field by an Agilent 3000 Micro GC with an error of ± 5%.

Data were displayed as the contour line maps obtained by computer-processed 'kringing' interpolation method in the SURFER 8.0 software package.

## 4. Results and discussion

### 4.1. Statistical analysis

**Figure 2:**
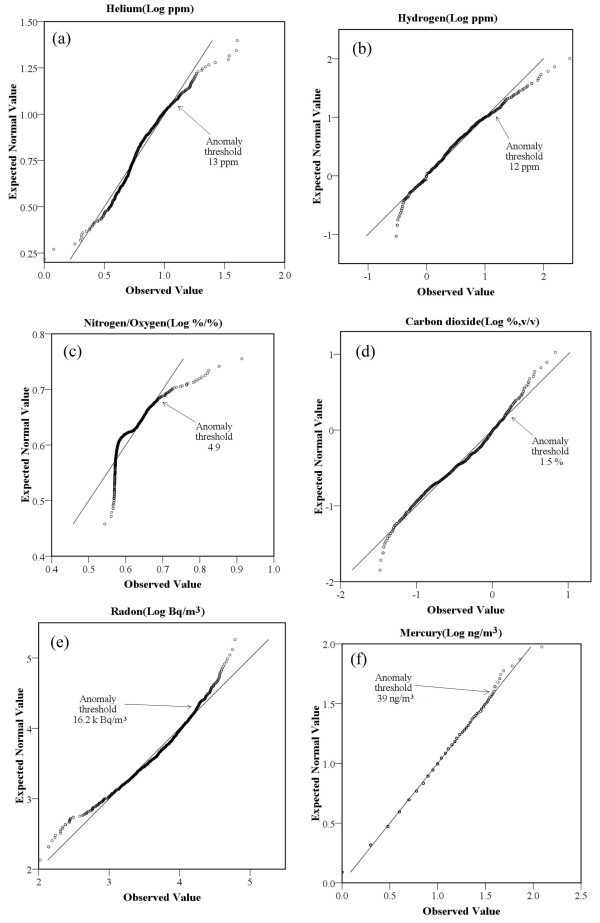
**The anomaly threshold of soil gas estimated by Quantile-quantile plots (Q-Q plot) method**. (a)He, (b) H_2_, (c) N_2_/O_2_, (d)CO_2_, (e)Rn, (f)Hg.

**Figure 3:**
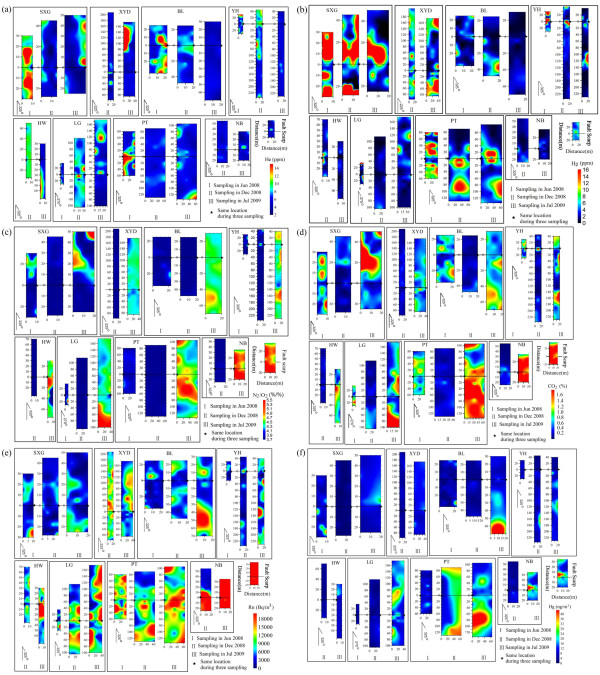
**Distribution of soil gas concentrations**. (a)He, (b) H_2_, (c) N_2_/O_2_, (d)CO_2_, (e)Rn, (f)Hg.

Statistical data for He, H_2_, CO_2_, N_2_, O_2_, Rn, and Hg concentrations in the soil gases are listed in Table 1. No detectable methane concentration was observed. Compared with the concentrations of components in air: Rn: 0.01 k Bq m^-3^, CO_2_: 0.036%, H_2_: 0.5 ppm, He: 5.2 ppm [14,15], the soil-gas samples exhibited much higher He, H_2_, CO_2_, and Rn concentrations. The Quantile-Quantile plot (Q-Q plot) provides a good method to distinguish different populations and to identify the background, anomalous values and extra outliers) [16], which is more objective to estimate statistically the anomaly threshold than the others [14]. The anomaly threshold values of soil gases along the co-seismic rupture zones were 13 ppm for He, 12 ppm for H_2_, 4.9 for N_2_/O_2_, 1.5% for CO_2_, 16.2 k Bq/m^3 ^for Rn, and 39 ng/m^3 ^for Hg (Figure 2). The spatial distributions of gas concentrations are displayed in Figure 3.

**Table 1:** The statistical data of soil gas concentrations in the seismic fault zone

Soil gas	Number of samples	Range	Mean	SD
He(ppm)	828	~40	8	4
H_2_(ppm)	828	~279.4	16	25
N_2_(%, v/v)	828	54.5~85.8	76.6	3.2
O_2_(%, v/v)	828	11.2~22.5	19.1	1.9
CO_2_(%, v/v)	828	0.03~6.70	0.63	0.6
Rn(k Bq/m^3^)	828	0.1~61.3	8.4	8.6
Hg(ng/m^3^)	828	0~122	9.4	9.3

The factor analysis results of the soil gas data showed the eigenvalues of two factors that exceeded one and contributed 55.2% of the total variance. Table 2 exhibits the loading of the matrix of varimax-rotated factor for the two-factor model. Factor 1 contributed 36.7% of the total variance, and had strong positive loadings on N_2_/O_2_, CO_2_, Rn, and Hg, which indicated they were controlled by similar geochemical processes. Factor 2 contributed 18.4% of the total variance and had strong positive loadings of He and H_2_, which are trace species in soil gas and may originate primarily from the deep earth.

**Table 2:** Loadings for Varimax-rotated factor matrix in two factor model

Soil gas	Components	
	
	1	2
He	-0.082	0.679713
H_2_	0.064	0.746152
N_2_/O_2_	0.885	-0.06386
CO2	0.868	0.069026
Rn	0.693	-0.034
Hg	0.396	-0.31721

### 4.2. The relationship between co-seismic rupture and soil gas

Anomalies of He, H_2_, CO_2_, Rn, Hg, and N_2_/O_2 _in soil gas displayed complex characteristics (Figure 3). The spatial distributions of soil gas concentrations clearly showed higher values near the fault scarps because co-seismic ruptures usually enhance the permeability of rock and soil, favoring gas emission and resulting in ''halo'' anomalies. At many sites, isolated points with high concentration values (''spotty anomalies'') were frequently observed [14]. The multiple ''spot anomalies'' along fault scarps probably mainly resulted from the differences in fault and soil features from place to place (Figure 3). The fault scarps observed upon the alluvial terrace, where unconsolidated deposits overlie basement rock, showed a complex morphology with a folding structure [1]. The bimodal feature of the anomalies of soil gas crossing the fault scarps may be caused by emission and atmospheric dilution of the soil gas on the fault scarps (Figure 3).

**Figure 4:**
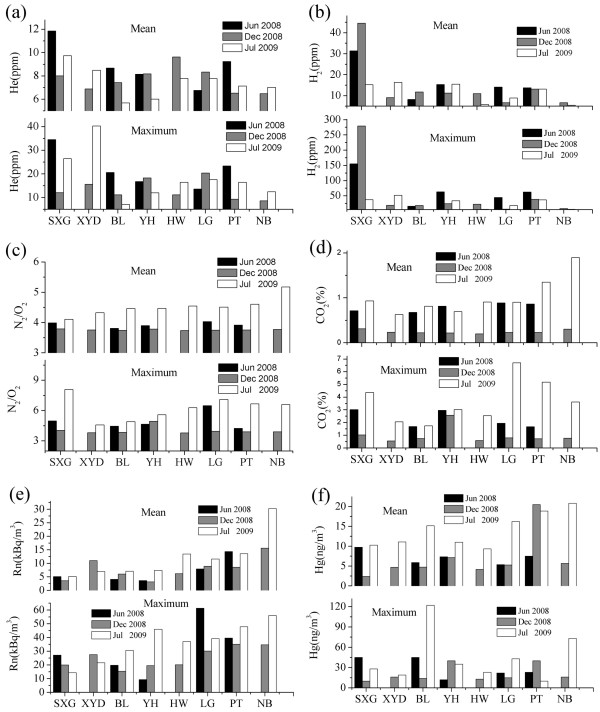
**The means and maximums of He and H_2 _concentrations**. (a)He, (b) H_2 _(c)N_2_/O_2_, (d)CO_2_, (e)Rn, (f)Hg.

**Figure 5:**
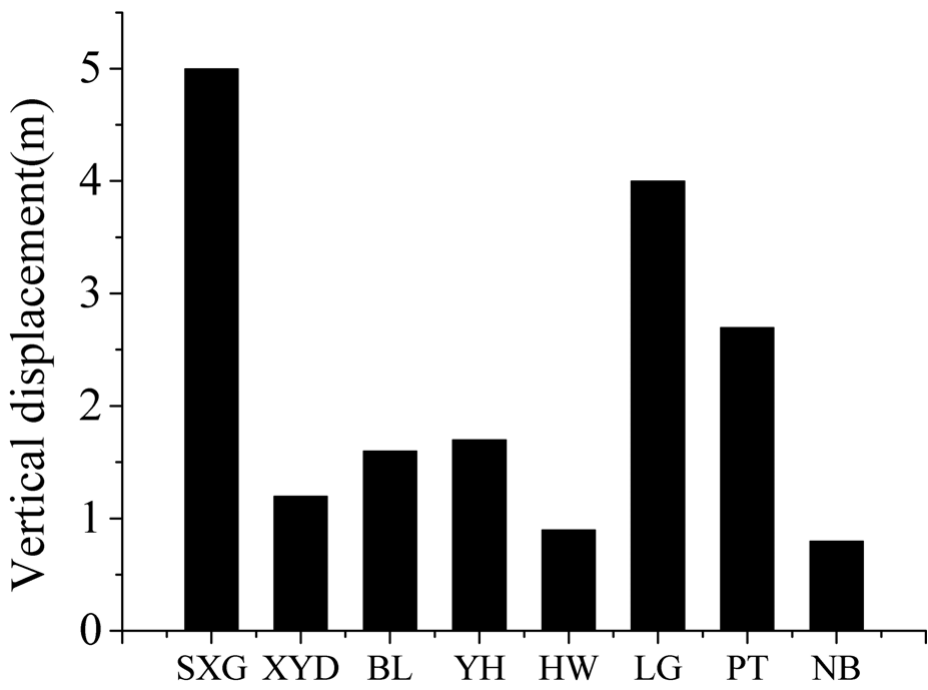
**Variations of vertical displacement along-strike at 8 sampling sites**.

The maximum concentrations of He and H_2 _(40 and 279.4 ppm, respectively) were found on all three sampling dates at the south part of the rupture zone close to the epicenter (Figure 4a and 4b). It is obvious that the maximum concentrations of He and H_2 _measured in July 2008 were positively correlated to the vertical displacement of fault scarp (Table 3). The maximum vertical displacement (5.0 m) of eight sites was at SXG along the Yingxiu-Beichuan fault zone (Figure 5). Helium migrates easily from deep accumulations (geothermal fluids, uraniferous ore, and hydrocarbon reservoirs) toward the surface along permeable fault and fracture systems, resulting in anomalies along the rupture zone [17]. Helium in spring waters in Longmenshan faults is predominately derived from the crust and mixes with mantle and atmospheric helium to a small degree [18]. H_2 _in soil gas might be produced by reaction between water and fresh mineral surfaces in the fault zone; such reaction depends upon the exposure of fresh rock surfaces via the episodic opening of cracks and fissures [19]. Highly variable H_2 _concentrations in space and time along seven active faults and around the aftershock region of the 2000 Tottori-ken Seibu earthquake in Japan were observed [20]. Additionally, the obvious low-velocity anomalies underlying the hypocenter region of the main shock suggested that gas-rich fluids derived from the lower crust might have intruded the vicinity of the hypocenter, which might be related to the earthquake and brittle failure of the upper crust [21].

**Table 3:** Correlative coefficient (CC) of mean and maximum concentrations of soil gas and vertical displacement of co-seismic rupture at eight sites from three measurements, respectively

	CC (Mean concentration)			CC (Maximum concentration)		
		
	Jun 2008	Dec 2008	Jul 2009	Jun 2008	Dec 2008	Jul 2009
He	0.45	0.12	0.55	0.53	0.29	0.17
H_2_	0.80	0.70	0.38	0.75	0.72	0.32
N_2_/O_2_	0.84	0.35	-0.55	0.54	0.04	0.73
CO_2_	0.08	0.38	-0.15	0.317	0.04	0.7
Rn	0.17	-0.37	-0.4	0.76	-0.11	-0.43
Hg	0.48	-0.001	-0.08	0.3	-0.11	-0.24

The distribution of Rn, CO_2_, and Hg concentrations in soil gas may be correlated primarily with the geological environment of fault rupture (Figure 3 Table 3). The decomposition of organic matter is an important source to soil gas CO_2_. CO_2 _in spring water at the Wenchuan Seismostation originated predominately from sedimentary organic matter [18]. These sources of CO_2 _can mask the information of mantle degassing in some places [9]. A generating mechanism of the Rn concentration anomalies in the co-seismic rupture was possibly enlargements of effective area of grain surface, porosity and radon emanation rates, which resulted from co-seismic cracking of rocks [22]. The soils above the bedrock always contain a certain amount of ^238^U and ^226^Ra, which means that there is a potential source of ^222^Rn in soil gas [23]. In the vapor phase, Hg is readily transported along permeable bedrock structures and through overlying soil cover. Therefore, higher values of Hg concentration occurred near the rupture zones. The background values of free mercury(^0^Hg) seemed to be more stable and the anomalous zones narrower than those of radon gas in the fault zone [24].

The concentrations of CO_2 _were obviously higher in the summer (June 2008, July 2009) than in the winter month (December 2008, Figure 4d). In July 2009, the increase of N_2_/O_2 _ratio was consistent with the CO_2 _concentrations at 8 sampling sites, which indicated that more CO_2 _was derived from lithosphere. The seasonal variations of CO_2 _concentrations in soil gas can be attributed to meteorological change, such as temperature, atmospheric pressure and humidity of air and soil [25]. However, the observations most likely reflect changes in microbial activities in response to subsurface temperature variations [26].

The similar distribution of N_2_/O_2_, CO_2_, and Rn anomalies, combined with the results of factor analysis, indicated the CO_2 _acted as a "carrier" for Rn (Figure 3). Rn has essentially a shallow origin due to its short half-life. The Rn concentration is never high enough for it to form a gas stream by itself, so a carrier gas is required [17]. In a wide range of geological settings, carrier gases (CO_2_, CH_4 _etc.) in the lithosphere may play a dominant role for no-diffusive transport and redistribution of trace gases (Rn, He and H_2_) toward the Earth's surface. However, some measured sites exhibited higher concentrations of carbon dioxide derived from biogenic sources, but no anomalies of other gases. The distribution of CO_2_, N_2 _and O_2 _in soil in the investigated area, was not matched well with He and H_2_, indicating He and H_2 _might migrate upwards by migration. In addition, the distribution of anomalous values of He and H_2 _matched well with the trace of the fault scarps, which may indicate that the crustal stress field severely affected the He and H_2 _transport in the fault zone.

### 4.3. The relationship between the aftershocks and soil gas

**Figure 6:**
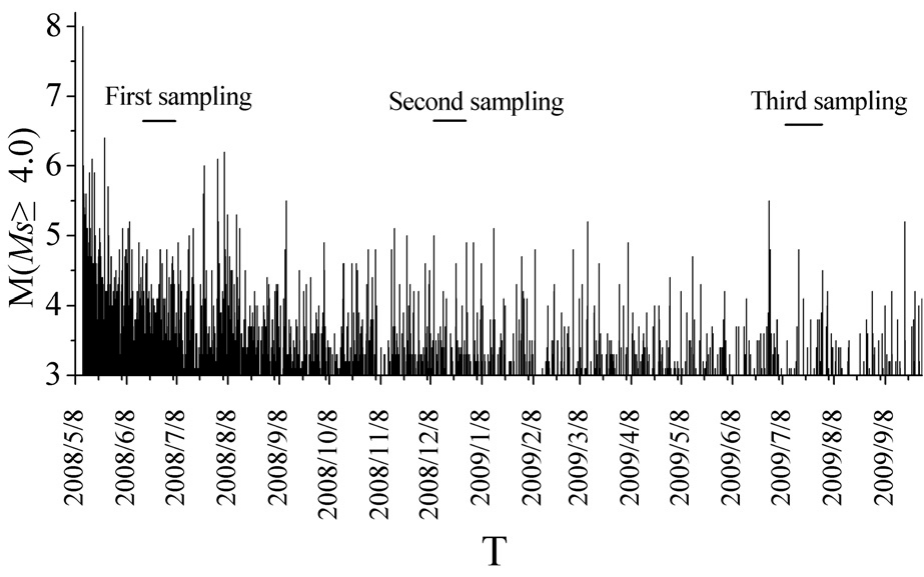
**M-T map**.

The results of soil-gas surveys in the fault zone after the Wenchuan *M*s 8.0 earthquake show that the spatial distribution of the gas anomalies were tectonically related to the seismic activity that enhanced gas leakage underground. The soil-gas anomalies varied with time, indicating a non-continuous leakage of the gases. A possible trigger of episodic leakage may be the aftershocks. 704 aftershocks with *M*_S _≥4.0 were recorded during 12 May 2008 to 30 September 2009 in the Longmenshan earthquake zone (Figure 6), of which 631 events are with magnitudes from *M*_S _4.0 to *M*_S _4.9, 64 from *M*_S _5.0 to *M*_S _5.9, and eight from *M*_S _6.0 to *M*_S _6.4. Increase of the regional stress field can cause change in fluid pore pressure, mobilizing various gases via advection along new faults and fractures or the reactivation of pre-existing brittle deformations [27]. Pressure gradients induced by stress change may cause a rapid migration of trapped soil gas. The phenomena that concentrations or fluxes of volatiles vary before and after earthquakes were observed [8].

The magnitudes of the He and H_2 _anomalies of three surveys declined significantly with time (Figure 3a and 3b). The higher concentrations of He and H_2 _in soil gas in the fault zone in June 2008 strongly indicated that the Wenchuan *M*s8.0 earthquake excited the release of gases from the fault zone (Figure 4a and 4b). Because crustal stress can accumulate at some part of the fault zone, helium emission can be enhanced. The spatial and temporal variations of helium anomalies, therefore, indicated variation of the stress field in the Longmenshan earthquake zone, as Zhu et al. [28] reported the intensity of helium emission could be related to the stress. The dramatic decrease in He and H_2 _concentrations in Jul 2009 may be due to reduction of permeability and porosity of rocks and soils in the rupture zone. Soil-gas He concentrations were higher in July 2009 at the XYD profile at which the co-seismogenic fault changes from reverse type to left-lateral strike-slip type.

The means of CO_2_, Rn and Hg concentrations were higher in the north parts than in the south parts of the co-seismic rupture (Figure 4d, e and 4f). Most of the large aftershocks were distributed in the north and south parts of the aftershock zone (Figure 1). The direction of the maximum principal stress gradually changed from near EW to near NW-SE along the main fault, and then turned to near EW at the utmost northern part of the main fault [29]. The higher values of radon counts were recorded as the sand column was stressed [30]. At the San Andreas Fault system between Santa Rosa and Cholame, the radon anomaly coincided reasonably well in time and space with larger local earthquakes above a threshold magnitude of about 4.0. These episodic radon changes may be caused by a changing outgassing rate in the fault zones in response to some episodic strain changes [31]. The hydromechanical modeling study of the Matsushiro earthquake swarm shows a clear connection between earthquake rupture, deformation, stress, and permeability changes, as well as large-scale fluid flow related to degassing of CO_2 _in the shallow seismogenic crust [32]. This extraordinary release of CO_2 _can cause a flash fluid pressure increase in the fault plane, and therefore, enhance earthquake slip or trigger aftershocks [33]. Kang et al. [34] found that the spatial features of mercury are correlated not only with the magnitude of earthquakes and distance from the epicenter to the observational site, but also with the stress field and medium between the focus and the observational site. Gu et al. [35] found that soil-gas Hg in the epicentral area decreased rapidly after the Shunyi 4.5 earthquake in China. The anomalies of CO_2_, Rn, and Hg concentrations could be related to the variation in the regional stress field and the aftershock activity.

## 5. Conclusions

The results obtained from this study led to the following conclusions:

1. The concentrations of He, H_2_, CO_2_, Rn, and Hg in the soil gas showed obvious anomalies near the fault scarps.

2. The maximum He and H_2 _concentrations in Jun 2008 occurred near the parts of the rupture zone where vertical displacements were larger. The distribution of Rn, CO_2_, and Hg concentrations in soil gas may be correlated with the geological environment of fault rupture and meteorological change.

3. The magnitudes of the He and H_2 _anomalies of three surveys declined significantly with decreasing strength of the aftershocks with time. The maximum concentrations of He and H_2 _were found three times at the south part of the rupture zone close to the epicenter. The spatio-temporal variations of CO_2_, Rn, and Hg concentrations differed obviously between the north and south parts of the fault zone. The anomalies of He, H_2_, CO_2_, Rn, and Hg concentrations could be related to the variation in the regional stress field and the aftershock activity.

## Competing interests

The authors declare that they have no competing interests.

## Authors' contributions

JD helped XZ write the manuscript. XZ, JD, ZC, JC, CX, YC, PY carried out the field-work and measured the concentrations of soil gas. JD, YT, LY, LY, LL, YL participated in the design and coordination of study. All authors read and approved the final manuscript.
